# Free-flow duration prior as an influential factor on microorganism and endotoxin amount of reverse osmosis water for dialysis

**DOI:** 10.1186/2047-2994-4-S1-P65

**Published:** 2015-06-16

**Authors:** Y Jao, SW Wu, TY Lin, CT Hung, PW Yang, CH Huang, CY Lin, WR Lin, YH Chen, PL Lu

**Affiliations:** 1 Department of Infection Control, Kaohsiung Medical University Hospital, Kaohsiung Medical University, Kaohsiung, Taiwan, Province of China; 2 Division of Infectious Diseases, Department of Internal Medicine, Kaohsiung Medical University Hospital, Kaohsiung Medical University, Kaohsiung, Taiwan, Province of China; 3 Clinical Microbiology Laboratory, Kaohsiung Medical University Hospital, Kaohsiung Medical University, Kaohsiung, Taiwan, Province of China

## Introduction

Reverse Osmosis water constitutes up to 99% of dialysis water that every time of hemodialysis requires about 150 liters. The microbiological and endotoxin monitoring of the water used for hemodialysis is extremely important in immunocompromised cases such as chronic kidney disease patients.

## Objectives

Although the frequency of RO water monitor is monthly, the detailed methods to collect the RO water was not clearly reported. We aimed to determine the duration of free-flow on the qualification rates of microorganisms and endotoxin in RO water.

## Methods

305 samples of dialysis water were collected from 61 collection sites at 8 medical units and 2 RO water producing facilities in a medical center. The water was collected for quality examination after 5 free-flow duration separately: 0, 5, 3, 5 and 10 minutes. The spread plate method was used to estimate colony count. The endotoxin was tested using Limulus Amebocyte Lysate (LAL) test. The qualification of colony count and endotoxin (EU) were defined as less than 100 CFU/ml and 0.25 EU/ml according to ISO 11663: 2009 regulation.

## Results

The colony count qualification rates in different free-flow duration were as following: 0 minute = 22.45%, 1 minute = 93.88%, 3 minutes = 93.88%, 5 minutes = 100%. After 10-minute free flow, the endotoxin qualification rate was only 61%. The result of endotoxin testing was normal after dialysis route being equipped with endotoxin filter. The qualification rates of colony count in storage water at RO water producing facilities were 75% after 0 minute and all 100% after one-minute duration. The endotoxin qualification rate were 0% of 0 minute, 0% of 1 minute, 75% of 3 minute, and 100% of 5 minute duration of free flow.

## Conclusion

In summary, we observed that colony count analysis for water collected after 5 minutes free flow or without free-flow will lead to indiscriminative result. Longer water route is associated with higher endotoxin disqualification rates. The discrepancy between the colony count testing and endotoxin analysis indicate the need for both testing to promote the quality of medical care and ensure the safety and health of dialysis patients.

## Disclosure of interest

None declared.

